# Laser Powder Bed Fusion of Copper–Tungsten Powders Manufactured by Milling or Co-Injection Atomization Process

**DOI:** 10.3390/ma17174394

**Published:** 2024-09-06

**Authors:** Simon Rauh, Shashank Deepak Prabhu, Gerhard Wolf, Lioba Fischer, Nico Hempel, Peter Mayr

**Affiliations:** 1 Fraunhofer UMSICHT, Fraunhofer Institute for Environmental, Safety and Energy Technology, An der Maxhütte 1, 92237 Sulzbach-Rosenberg, Germany; shashank.prabhu@umsicht.fraunhofer.de (S.D.P.); gerhard.wolf@umsicht.fraunhofer.de (G.W.);; 2 Chair of Materials Engineering of Additive Manufacturing, TUM School of Engineering and Design, Technical University of Munich, Freisinger Landstraße 52, 85748 Garching, Germany; nico.hempel@tum.de (N.H.); peter.mayr@tum.de (P.M.); 3 Faculty of Mechanical Engineering, University of Applied Sciences Ingolstadt, Esplanade 10, 85049 Ingolstadt, Germany

**Keywords:** laser powder bed fusion, atomization, copper–tungsten, composites, reflectivity, absorptivity, porosity, relative density, thermal conductivity, electrical conductivity

## Abstract

The processing of pure copper (Cu) has been a challenge for laser-based additive manufacturing for many years since copper powders have a high reflectivity of up to 83% of electromagnetic radiation at a wavelength of 1070 nm. In this study, Cu particles were coated with sub-micrometer tungsten (W) particles to increase the laser beam absorptivity. The coated powders were processed by powder bed fusion-laser beam for metals (PBF-LB/M) with a conventional laser system of <300 watts laser power and a wavelength of 1070 nm. Two different powder manufacturing routes were developed. The first manufacturing route was gas atomization combined with a milling process by a planetary mill. The second manufacturing method was gas atomization with particle co-injection, where a separate W particle jet was sprayed into the atomized Cu jet. As part of the investigations, an extensive characterization of powder and additively manufactured test specimens was carried out. The specimens of Cu/W powders manufactured by the milling process have shown superior results. The laser absorptivity of the Cu/W powder was increased from 22.5% (pure Cu powder) to up to 71.6% for powders with 3 vol% W. In addition, a relative density of test specimens up to 98.2% (optically) and 95.6% (Archimedes) was reached. Furthermore, thermal conductivity was measured by laser flash analysis (LFA) and thermo-optical measurement (TOM). By using eddy current measurement, the electrical conductivity was analyzed. In comparison to the Cu reference, a thermal conductivity of 88.9% and an electrical conductivity of 85.8% were determined. Moreover, the Vickers hardness was measured. The effect of porosity on conductivity properties and hardness was investigated and showed a linear correlation. Finally, a demonstrator was built in which a wall thickness of down to 200 µm was achieved. This demonstrates that the Cu/W composite can be used for heat exchangers, heat sinks, and coils.

## 1. Introduction

Powder bed fusion-laser beam for metals (PBF-LB/M) is an additive manufacturing (AM) method to produce complex component geometries with high precision. The range of processable materials, as well as their qualification for the PBF-LB/M process, is continuously being further developed. In recent years, one research focus in the field of AM has been the processing of copper (Cu). The industry shows enormous interest in realizing AM of Cu. The excellent properties of Cu, such as its high thermal conductivity, electrical conductivity, ductility, and corrosion resistance, are reasons behind its indispensability in the fields of electronics and thermal applications. However, the reflectivity of 83% at a wavelength of 1070 nm [1] for neodymium-doped yttrium aluminum garnet (Nd:YAG) lasers poses challenges in processing Cu by conventional PBF-LB/M machines with a laser power of <300 watts. Due to the high reflectivity of the Cu powder, the energy of the laser beam is insufficiently absorbed, resulting in an incomplete melting of the powder. Furthermore, due to the high thermal conductivity of the Cu, the generated thermal energy is quickly dissipated and the melt pool cools down rapidly. Both phenomena lead to an increase in porosity.

Research in this field describes various options for producing dense Cu components via the PBF-LB/M process. For example, increasing energy density by decreasing the spot diameter and/or increasing the laser power [2,3,4]. Another approach is the use of lower wavelengths, i.e., a “green” (wavelength of 515 nm) or “blue” (wavelength of 450 nm) laser [5,6,7]. Further options are the alloying of Cu with additives [8,9,10,11], which cause a reduction in thermal conductivity and especially of electrical conductivity. Finally, there is also the solution of coating the Cu powder particles to increase the absorptivity of the laser energy [12,13,14,15]. A few publications discuss the influence of W content on Cu/W composite powders [1,16]. They describe that W increases the absorptivity of the electromagnetic radiation of the laser beam. The main reason for using W is its solid insolubility in Cu [17]. W potentially has only a minor effect on the electrical and thermal conductivity in a Cu/W building part because it does not affect the conductivity properties of the Cu matrix.

There are several studies which describe the processing of Cu/W composite by the PBF-LB/M process. Gu et al. [18] manufactured test specimens of W-20 wt% Cu by using a 2000-watt laser with a relative density of up to 95%. By using a 100-watt laser, Li et al. [19] fabricated test specimens of W-10 wt% Cu and they measured a relative density of up to 66%. For increasing the relative density of Cu/W composites, nickel (Ni) is used, but it reduces the conductivity properties. A limited number of publications describe the effect of Ni in additively manufactured Cu/W composites [20,21,22]. Yan et al. [23] processed various compositions of Cu/W composites with Cu contents of 15–40 wt%. They worked with a 200-watt laser system and achieved relative densities of up to 98%. As part of their investigations, they also analyzed thermal conductivity, thermal expansion, roughness, and hardness. Furthermore, Zheng et al. [1] coated Cu particles with a mass fraction of 0.5 wt% W particles. For this purpose, an innovative chemical coating process was developed by using Cu powder suspended in an ammonium W solution. W oxides form on the Cu particle surface, which is reduced to metallic W by calcination. As a result, the surface of the Cu particles was homogeneously covered with very fine W particles without deforming the Cu particles. With a 500-watt laser, they achieved a relative density of 98.5% and an electrical conductivity of 96%, according to the International Annealed Copper Standard (IACS).

In the present study, the Cu particle surface was coated with sub-micrometer tungsten (W) particles. Compared to the publications mentioned above, two different production methods of metal matrix composite (MMC) powders are described in this work. A coating process via a milling process and a co-injection atomization process were chosen to produce the Cu/W composite powders. The aim is to develop a solution to process Cu for conventional systems with a laser power of <300 watts, which is already in use in the industry. Thereby, the coating processes should increase the absorptivity of the laser beam on the Cu particles and maintain the conductivity properties. The investigations include an extensive material characterization as well as the manufacturing of a demonstrator that illustrates the feasibility of the wall thicknesses.

## 2. Materials and Processes

### 2.1. Powder Manufacturing by Gas Atomization

To produce the Cu powder, Cu ingots were melted by induction from a coil in a graphite crucible under a nitrogen (N_2_) atmosphere (nitrogen 5.0, Linde, Dublin, Ireland). Due to gravity and suction, the melt was pressed through an outlet nozzle. The resulting melt jet was atomized directly at outlet ports by a high-pressure gas stream of N_2_ gas. The atomization temperature was 1150 °C. The resulting powder particles were cooled with liquid N_2_ (liquid nitrogen, Air Liquide, Paris, France). Two different fractions were produced. A finer fraction was separated by a cyclone and a coarser fraction was collected in a container at the bottom of the atomization chamber. The powder containers were sealed and removed from the atomization plant. The containers were channeled in a glove box (MB 150-G-I, MBraun, Garching bei München, Germany). The produced powder was classified with a 32 μm sieve (Fritsch, Idar-Oberstein, Germany) inside the glove box. The sieving was carried out under an N_2_ atmosphere. Powder samples were taken to determine the grain size distribution. Laser diffraction analysis (Lasergranulometer HELOS/BR, Sympatec, Clausthal-Zellerfeld, Germany) was used to measure the particle size distribution. To ensure high purity and low oxygen concentration, the powder production was carried out in an inert atmosphere.

### 2.2. Composite Powder Manufacturing by a Milling Process

The W particles (Wolfram Bergbau und Hütten AG, St. Martin, Austria) were incorporated into the surface of the Cu particles by means of a kinetic grinding process using a planetary ball mill (Pulverisette 5/4, Fritsch). In the glovebox, a powder mixture of 93.74 wt% Cu and 6.26 wt% W was weighed in, which is equivalent to the volume fractions of 97% Cu and 3% W. The Cu powder had a measured mean particle size (d_50_) of 25.5 μm. According to the powder manufacturer, the W powder had a d_50_ of 0.7 µm. The milling container and milling balls were made of hardened steel. During the milling process, heat was released by the high kinetic energy. The high temperature, combined with the kinetic energy, caused the deformation of the Cu particles. In this respect, the grinding process was optimized in terms of the speed and milling time for achieving a maximum of W bonding on the Cu particles at the lowest rate of particle deformation. After the grinding process, the powder was analyzed by laser diffraction analysis, scanning electron microscopy (1450 VP, LEO/Zeiss, Oberkochen, Germany), and X-ray fluorescence analysis (S1 Titan, Bruker, Billerica, MA, USA). Additionally, energy-dispersive X-ray spectroscopy (INCA PentaFETx3, Oxford Instruments, Abingdon, UK) and inductively coupled plasma optical emission spectroscopy (5800 ICP-OES, Agilent Technologies, Santa Clara, CA, USA) were used to detect possible impurities.

### 2.3. Composite Powder Manufacturing by Co-Injection Atomization

**Figure 1:**
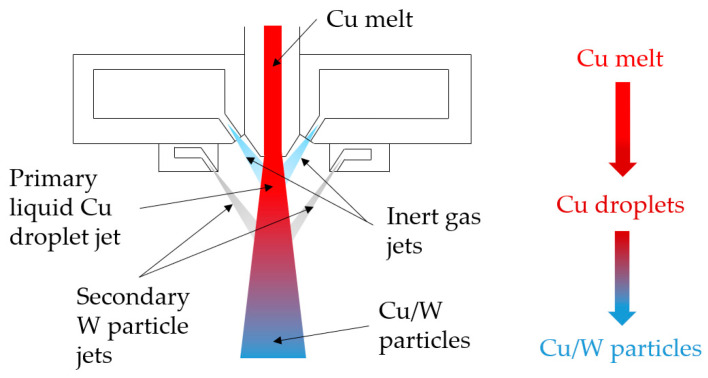
Schematic structure of the co-injection atomization process.

The co-injection atomization process is related to the gas atomization method as described in Section 2.1. In addition to the nozzle and gas openings, injection pipes were installed. Sub-micrometer W particles with a d_50_ of 0.7 μm were sprayed into the melted powder particle jet through the injection pipes using a powder conveyor. The co-injection atomization process is shown in Figure 1. Due to the manufacturing process, it was possible to produce highly spherical composite powder particles in situ without any further process steps. Two batches of co-injection powder were produced with different W content by adjusting the flow rate of the conveyor. The chemical composition was measured by an X-ray fluorescence analysis (XRF).

### 2.4. Powder Bed Fusion-Laser Beam

In this investigation, the process parameters of the PBF-LB/M system (ProX DMP 200, 3D Systems, Rock Hill, SC, USA) were optimized for the powder materials used, i.e., the pure Cu and the Cu/W composites. The aim was to produce specimens with the highest possible relative density. For this purpose, the laser power and scanning speed were varied. The laser source was a Nd:YAG laser with a wavelength of 1070 nm and a maximum laser power output of 262 watts. Finally, a laser power of 249 watts and 262 watts was used. The optimized laser scanning velocity was 850 mm/s. The hatch distance was 60 μm and the layer thickness was 30 μm. Therefore, the volume energy density was 171 J/mm^3^. An argon (Ar) atmosphere (argon 4.6, Linde) was used in the process chamber. During the manufacturing process, the oxygen (O_2_) content was kept at 700 ppm. The build plates on which the samples were created were made of steel (1.4104).

### 2.5. Assembly for Powder Supply and Build Chamber with Space Reduction

**Figure 2:**
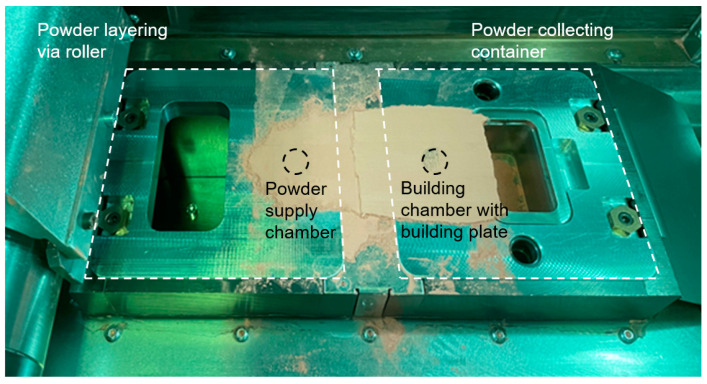
PBF-LB/M process chamber with a special assembly for reduced amounts of powder supply and build chamber size.

The build chamber has the dimensions of 140 × 140 × 100 mm^3^. As shown in Figure 2, the installation of a piston reducer (3D System) has reduced the volume of the powder supply chamber and the build chamber enormously. The chamber geometry is cylindrical and has a diameter of 20.25 mm. The height of the powder feeding chamber is 65.5 mm, with a volume of 21 cm^3^. This application made it possible to reduce the amount of powder used to 120 mL. Without the piston reducer assembly, the average powder consumption in the construction of test specimens for a parameter study is 1.5 L filling quantity.

For the material characterization, test specimens of cylindrical geometry with a diameter of 13 mm and a height of 3 mm were specified. The additively manufactured samples had a height of 20 mm and a diameter of 13 mm. From the cylindrical geometry, further test specimens of about 5 mm height were prepared, which were ground and polished to a height of 3 mm. Two milling process samples, two co-injection atomization samples, and one pure Cu sample were additively manufactured. The milling process samples were made from the same Cu/W powder with different laser power (P_milling process 1_ = 249 watts; P_milling process 2_ = 262 watts). The two co-injection atomization samples were manufactured by two co-injection atomization powders with similar process parameters.

## 3. Characterization Methods

### 3.1. Powder Flowability and Density Measurements

The flowability of the powders was measured by a hall flowmeter according to DIN EN ISO 4490 [24]. The measurements were carried out by means of a calibrated funnel. The apparent density (DIN EN ISO 3923-1) [25] and tap density (DIN EN ISO 3953) [26] were also measured via the hall flowmeter funnel.

### 3.2. Reflection Measurement of Electromagnetic Radiation

The spectrophotometer (Lambda 950, PerkinElmer, Waltham, MA, USA) was used to measure the reflectivity of electromagnetic radiation. An integrating sphere was equipped, which measured reflections in a wavelength spectrum from 175 to 3300 nm. This allowed measuring the reflection of the pure Cu and W powders and the Cu/W powder mixtures and thus determined the absorption of the laser beam at a wavelength of 1070 nm. The powders were characterized in a wavelength range from 900 to 1200 nm.

### 3.3. Optical Porosity Measurement

**Figure 3:**
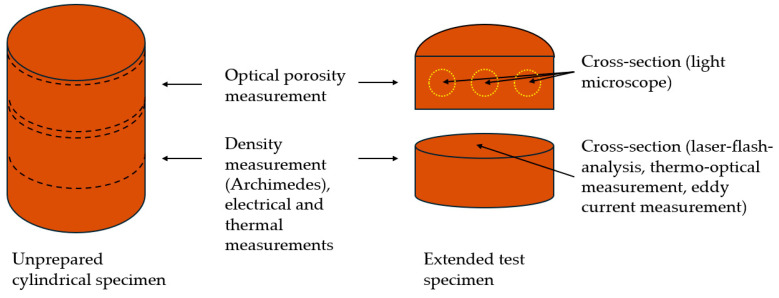
Schematic illustration of the extraction of the samples and cross-sections for each analysis method.

The vertical sections of the test specimens were analyzed using a light microscope to determine porosity (Eclipse LV 150, Nikon, Tokyo, Japan). The samples were cut, embedded, ground, and polished. The porosity was then analyzed at 100× magnification. Figure 3 depicts the examined cross-section. It was measured at three locations per sample, and the mean value was calculated. The results were used to adjust the process parameters and minimize porosity.

Figure 3 shows the extraction of the samples and the analyzed cross-sections of the optical porosity and density measurement (Archimedes), as well as the electrical and thermal analyses. The electrical and thermal analyses are described in Section 3.6, Section 3.7 and Section 3.8.

The optical porosity was evaluated using an image analysis software (dhs Bilddatenbank V14, Dietermann & Heuser Solution GmbH, Greifenstein-Beilstein, Germany). The microstructure image is automatically converted into gray values and binarized to calculate the porosity. By using a defined threshold value or by selecting directly in the spectral histogram, the gray value range of the image can be controlled [27].

### 3.4. Density Measurement According to the Archimedean Principle

The relative densities of the samples were measured with a precision balance (ABJ 320-4NM, KERN & SOHN, Balingen, Germany) and with a universal density determination set (YDB-03, KERN & SOHN) in air and ethanol (VWR, Radnor, PA, USA). The ethanol content was 99.5% according to the data sheet. The measurements were carried out at a temperature of 20.5 °C. A density of 8.92 g/cm^3^ was assumed for the Cu samples. The density of the Cu/W sample was then calculated according to Equation (1):

(1) $$\rho_{Cu/W}=\frac{\rho_{Cu}\cdot \;x_{Cu}+\rho_{W}\cdot x_{W}}{100}$$

ρ = density.

x = volume percent.

The density values ρ_Cu_ = 8.92 g/cm^3^ and ρ_W_ = 19.25 g/cm^3^ have been taken from the literature [28,29]. The volume fractions x_i_ of the Cu/W milling process samples were taken from the weight calculation, while the x_i_ of the Cu/W co-injection samples were determined based on the results of the X-ray fluorescence analysis (XRF) as an exact chemical composition could not be ensured during the co-injection atomization process. Thus, theoretical densities of 9.23 g/cm^3^ for the milled powder, 9.08 g/cm^3^ for the co-injection powder (1), and 9.03 g/cm^3^ for the co-injection powder (2) were determined.

### 3.5. Microstructure and Surface Morphology Analysis

The surface structure of the test specimens was analyzed using a digital microscope (VHX 2000, Keyence, Osaka, Japan). The images of the microstructures of the polished cross-sections were taken with a digital microscope and scanning electron microscope (JSM-6490, JEOL, Tokyo, Japan).

### 3.6. Laser Flash Analysis (LFA)

The thermal diffusivity was measured by a laser flash analysis (LFA 457 MicroFlash, Netzsch, Selb, Germany). A Nd:glass short-pulse laser with a max. pulse energy of 18 J, a wavelength of 1.06 μm, and a pulse width of 0.3 ms was used to pulse onto the polished sample surface. The specimen was coated with a thin layer of graphite to reduce the laser pulse reflectivity and increase the absorption of the laser pulses. On the opposite side of the sample, the temperature distribution was measured simultaneously by an infrared detector. The thermal diffusivity was determined from the temporal temperature distribution and the number of pulses. The thermal conductivity was calculated according to Equation (2) [30].

(2) $$\lambda (T)=a(T)\;\cdot \;c_{p}(T)\cdot \;\rho (T)$$

λ = thermal conductivity.

a = thermal diffusivity.

c_p_ = specific heat capacity.

ρ = density.

The specific heat capacity c_p_ and density ρ as a function of temperature were calculated by an interpolation of the literature values [29,31,32]. A 4th-grade polynomial function was chosen for the calculation of the specific heat capacity. To calculate the specific density, a volume-related linear function was used.

### 3.7. Thermo-Optical Measurement (TOM)

Thermo-optical measurement can be used to determine optical and thermal material properties and was initially developed for the observation of sintering processes. These encompass thermal diffusivity, heat capacity, thermal conductivity, the transmission of thermal radiation, and the scattering of light. In addition, thermal expansion can be measured using an optical dilatometer. Heat diffusivity is measured using the laser flash method [33]. Due to the deviation of the results for heat diffusivity from Netzsch’s LFA at the start temperature of 25 °C, the TOM was used as a comparative measurement. The performed laser flash analysis of TOM consisted of 15 separate measurements of the thermal diffusivity at a constant temperature, from which the mean value was determined.

### 3.8. Eddy Current Measurement

The electrical conductivity was determined according to ASTM E1004 [34] by using eddy current measurement (SIGMASCOPE^®^ SMP350, Helmut Fischer GmbH, Sindelfingen, Germany), which is a phase-sensitive method. The probe consists of a ferrite core surrounded by two coils. A high-frequency magnetic field is generated by means of the excitation coil, which generates eddy currents in the sample. The measuring coil measures impedance which is influenced by the eddy currents in the sample and out of phase by an angle φ relative to the excitation current [35].

### 3.9. Hardness Measurement

The hardness was determined according to the Vickers test (Micromet 5103, Buehler, Lake Bluff, IL, USA). A test weight of 1 kg (HV1) was used. The measurements were carried out in accordance with DIN EN ISO 6507-1 [36]. All the specimens were measured in a polished condition. The hardness of each sample was measured at three locations and the mean value was calculated.

## 4. Results and Discussion

### 4.1. Morphology of Powder Particles

**Figure 4:**
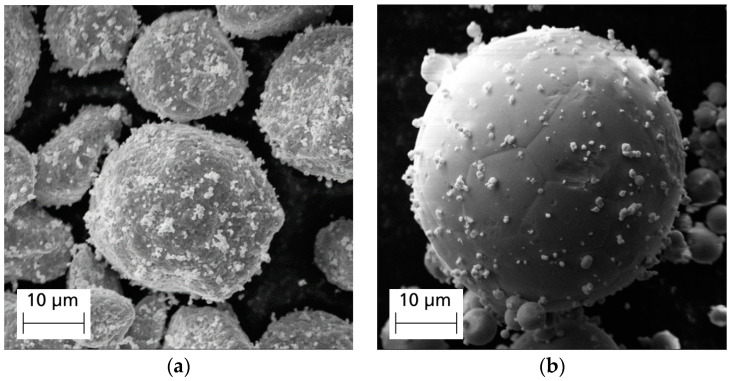
SEM images of produced Cu/W powders before sieving. (**a**) Milling process powder 1; (**b**) Co-injection powder 1.

In Figure 4, the Cu/W powders produced by different manufacturing routes are shown. Image (a) shows the powder particles produced in the mechanical milling process, whereas image (b) shows a powder particle manufactured by the co-injection process.

After the mechanical milling, the Cu particles are coated with a large number of small W particles. During the process, the Cu particles are slightly deformed due to the kinetic energy and the resulting elevated temperature. Also, the Cu particles may partially grow through agglomeration and fusion effects. Therefore, the modified powder is sieved using a 32 μm sieve after the milling process in order to remove coarser or deformed Cu particles. To determine the relative density at higher precision, the chemical composition is measured by XRF, as described in Section 4.3 and Section 4.7. Furthermore, irregular particle shapes have a negative effect on powder flowability, which has been investigated in many publications [37,38]. Moreover, the surface roughness can reduce the flowability and thus have a negative impact on processability [39]. In contrast, the particle shapes of the co-injection powders are highly spherical and the amount of W particles on the Cu surface is considerably lower than that of the milling process powder. Consequently, the surface of the Cu particles is less rough and deformed and those powders should be less affected by decreasing flowability phenomena. Another benefit of the co-injection powder is that it can be processed in a single manufacturing step directly during atomization and the risk of contamination with impurities, which is always given in milling processes, can be avoided.

### 4.2. Flowability, Apparent Density, and Tap Density of the Powders

The flowability measurement according to DIN EN ISO 4490 [24] shows that the powders <32 µm are non-flowable. The particle size plays a key role in the rheological behavior of the powder. A large amount of fine powder particles increases inter-particle friction, which affects the flowability [37]. The morphology and surface roughness of the particles also influence the rheological behavior of the powder. The spherical and smooth particles form a denser powder bed, which leads to better component properties in the PBF-LB/M process [37,38]. Nevertheless, the used powder feeding system with a roller is designed for processing very fine powders with poor flowability.

The measured apparent density ρ_ac_ and tap densities ρ_t_ of the processed powders are shown in Table 1. ρ_ac_ and ρ_t_ increase with the W content. The reason is that the W particles are mainly located on the surface of the Cu particles and form limited W agglomerates. This allows them to increase ρ_ac_ and ρ_t_ with their mass volumetric density. In Section 4.3, the W content of the powders is measured.

The extremely fine W powder <0.7 µm has the lowest ρ_ac_ and ρ_t_. A fine powder has an increased number of voids, so apparent and tap densities are decreasing.

### 4.3. X-ray Fluorescence Analysis of the Powders

Table 2 shows the measured weight percentages of Cu, W, and impurities. An increased percentage of impurities was measured in the powder produced by the mechanical milling process.

In the milling process powder, 2.19 wt% silicon (Si) was detected, which could not contaminate the powder during the milling process because 1.18 wt% Si was also detected in the co-injection powder 2. The measurements were carried out several times with different calibrations, whereby the Si content was always detected. In order to exclude the Si contamination of the powders, the measurements were also carried out using energy-dispersive X-ray spectroscopy (EDX). However, no Si was detected in any of the powder samples. Additionally, inductively coupled plasma optical emission spectroscopy (ICP-OES) was performed to detect Si impurities. The powder samples of the Cu/W milling process, Cu/W co-injection atomization 1, and Cu/W co-injection atomization 2 were analyzed and only traces of Si of 78–200 ppm were detected. The other impurities were either already in the raw material or were added during the atomization process. Furthermore, there is a measurement inaccuracy of the XRF, which is discussed in the comparative measurements on the additively manufactured cylindrical samples in Section 4.7.

### 4.4. Spectrophotometry

**Figure 5:**
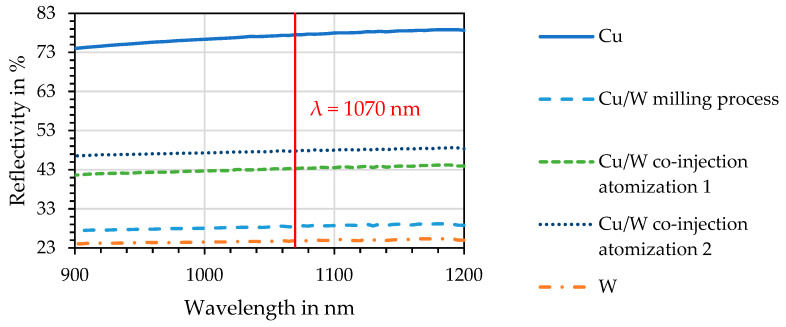
Reflectivity is plotted against the wavelength.

Figure 5 depicts the reflection as a function of the wavelength. Pure Cu powder has by far the highest measured reflectivity of 77.5% among all the compared specimens. This means that it absorbs only 22.5% of the electromagnetic radiation of the laser beam at a wavelength of 1070 nm.

The co-injection atomization process allows the coating of the W particles to the surface of the Cu particles and thus reduces the reflectivity of the co-injection powder (1) to 43.3% and of the co-injection powder (2) to 47.8%. The lowest reflectivity values of 28.4% were achieved for the milling process powder. This means that during the PBF-LB/M process, 71.6% of the radiation is absorbed by the Cu/W milling process powder. Since the pure W powder only has a slightly lower reflectivity of 24.9%, it can be inferred that a high percentage of the surface of the Cu particles is covered with the W particles by the milling process, which contributes to the increased absorption of incident radiation. The co-injection powders can reduce the reflection to a smaller extent, as the W content in the powder is lower compared to the milling powder. This is also shown by the results of the XRF analysis. Further important factors influencing the measured reflectivity are the morphology and surface roughness of the particles. These cause the scattering of electromagnetic radiation and thus also increase the absorptivity.

**Figure 6:**
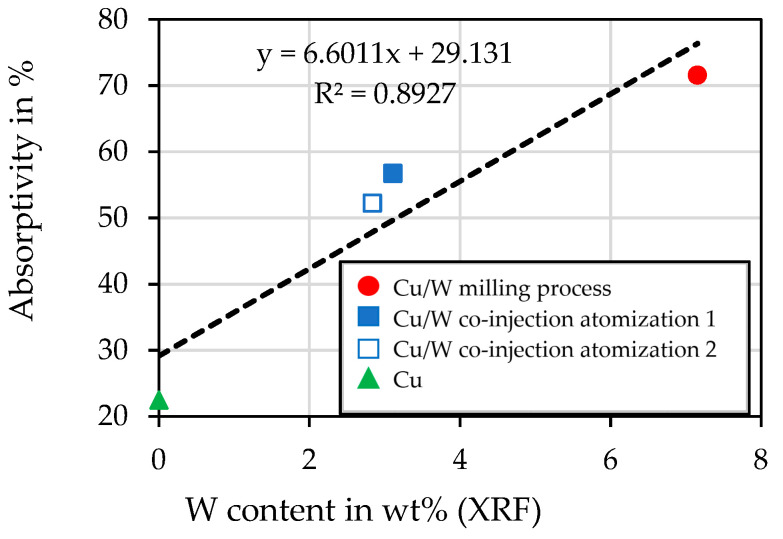
The influence of W content (XRF) on the absorptivity measured by spectrophotometry.

Figure 6 shows the dependency of absorptivity measured for the powders on their W content. This confirms an increase in the absorptivity of the Cu powder through the coating of the W particles. By adding 3 vol% of W, the absorptivity can be increased by a factor of 3.2.

### 4.5. Cylindrical Test Specimens

**Figure 7:**
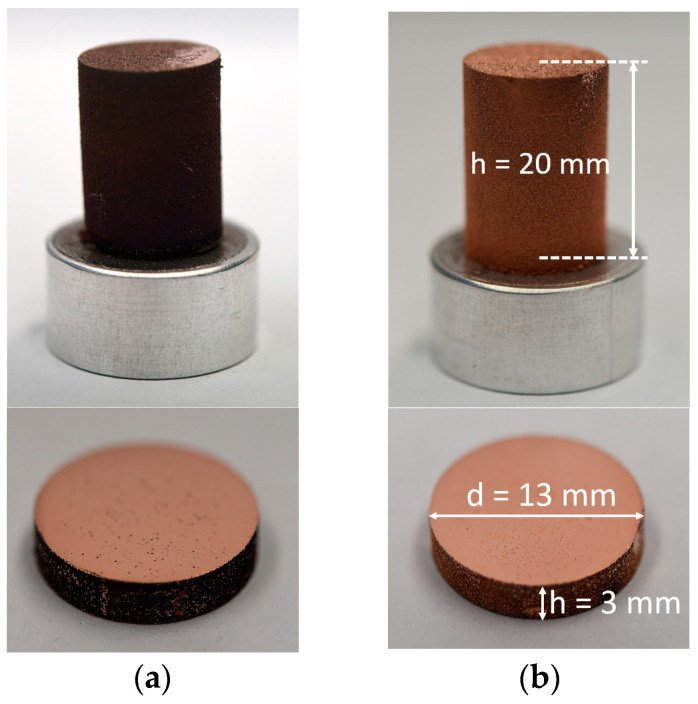
Macro images of the additively manufactured samples and their extracted cross-sections. (**a**) milling process 2; (**b**) co-injection process 2.

Figure 7 shows the macro images of the test specimens, which are either the unprocessed, additively manufactured, or extracted cross-sections of the test specimens.

The sample from the milling process was damaged by the roller at the end of the PBF-LB/M process and therefore, it was slightly tilted on the build plate. Despite this, the process was completed successfully. This singularity is irrelevant for further investigations since the cylindrical sample for further investigation has been cut from the center area of the cylinder.

### 4.6. Surface Morphology and Microstructures

#### 4.6.1. Digital Microscopy

**Figure 8:**
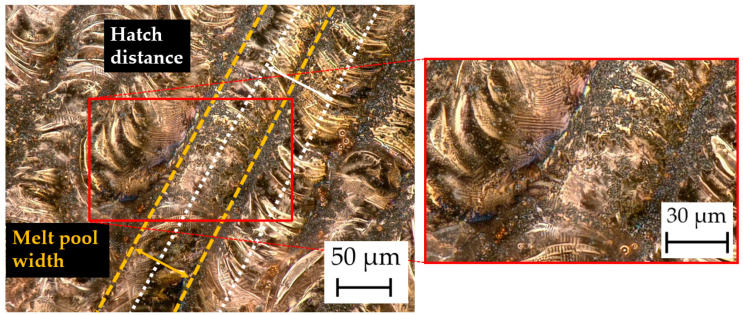
Surface morphology of Cu/W co-injection 1 compound.

Figure 8 shows the analyzed surface of the manufactured samples. The solidified melt pools are visible. The hatch distance and effective zone of the laser beam (melt pool width) are depicted in the images.

In the magnified image of Figure 8, the fine W particles (dark gray spots) are visible. Despite the higher density of W, it appears to float on the surface of the melt pool due to the high surface tension of the liquid Cu. This can lead to a heterogeneous distribution of W in the Cu matrix in the MMC. Also, the Marangoni effect, which describes the convection flows in the melt pool, may not be sufficient to homogenously disperse the W particles in the Cu melt [40].

**Figure 9:**
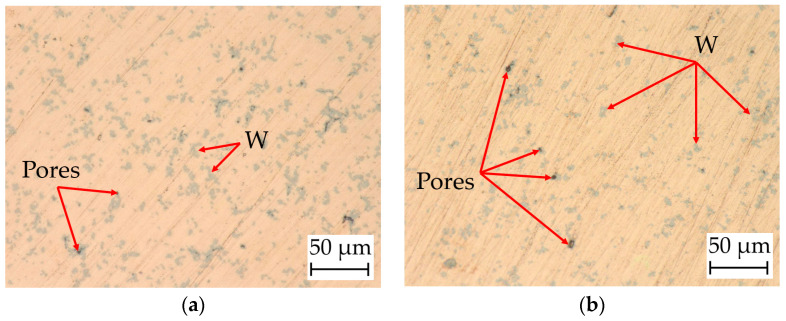

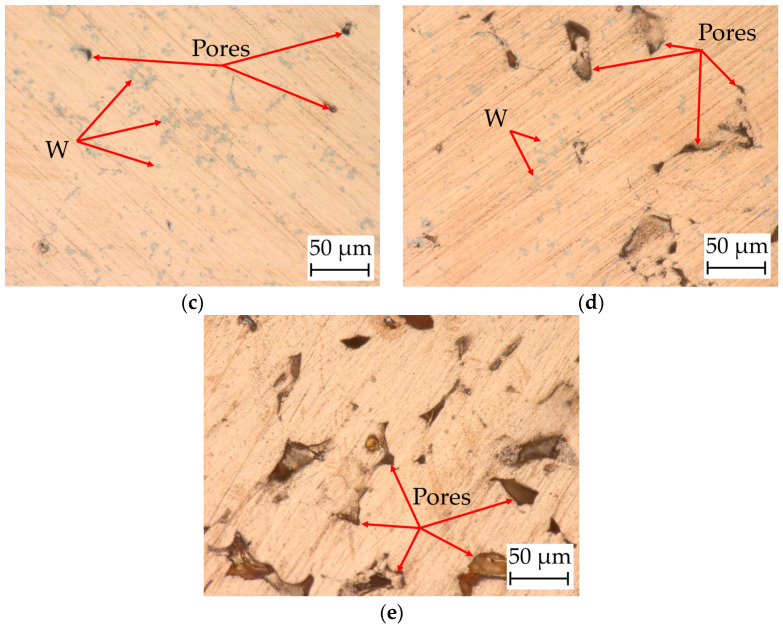
Microstructures of additively manufactured Cu and Cu/W composite test specimens. (**a**) Cu/W milling process 1; (**b**) Cu/W milling process 2; (**c**) Cu/W co-injection atomization 1; (**d**) Cu/W co-injection atomization 2; (**e**) pure Cu.

Figure 9 shows the microstructures of the additively manufactured Cu and Cu/W composites. The selected areas demonstrate the microstructural evolution of the specimens in largely undisturbed areas and do not give a representative overview of the pore sizes and distribution of the whole samples. The images depict the Cu matrix, elementary W zone (gray), and pores (dark gray and black). The W particles are not homogeneously distributed in the Cu matrices and form, to a certain extent, agglomerates. Figure 9a,b exhibit only small pores (black spots), which are mainly located in the center of the W agglomerate zones.

This indicates that pores remain between the non-melted W particle–particle contacts where the Cu melt cannot infiltrate. Such W zones partially also seem not to be completely surrounded by the Cu matrix. Figure 9c,d of the co-injection atomization specimens exhibit an increased size of the pores. The number of zones with W agglomerates is reduced compared to the milling process specimens, but it is still visible. The pore sizes increase with decreasing W content. While a huge portion of the porosity in specimens 9(a) to 9(c) is sub-micron or only a few microns in size, considerably larger pores occur in the specimen with the lowest W content (Figure 9d). Here, the pore structures become similar to the ones of the specimen generated with pure Cu (Figure 9e). For pure Cu, a lack of fusion is obvious. These pores reach dimensions larger than 50 µm. Predominantly large pores are visible due to a lack of fusion for the additively manufactured Cu and the low-W specimens (2.3 wt% W) as well as very fine pores above a W content of 3.4 wt% W. The fine pores are predominantly located inside or close to the W particles and agglomerates. Consequently, such pores may originate either from a Cu vapor formation or, more probably, due to a shielding effect of the non-melting W particles that foster gas inclusions.

#### 4.6.2. Scanning Microscopy

**Figure 10:**
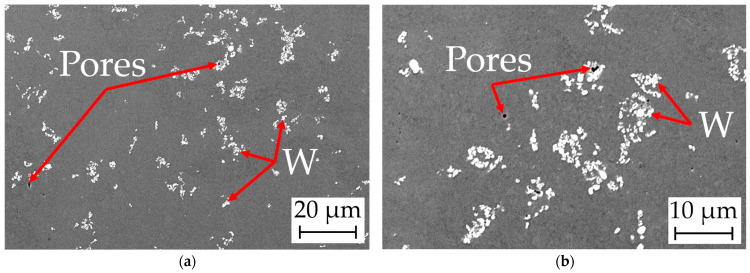
SEM images of the microstructure of the Cu/W milling process 1 sample (**a**,**b**) at different magnifications.

The microstructure analyzed by scanning electron microscopy is shown in Figure 10. The white spots are the W particles and agglomerate zones surrounded by the dark gray Cu matrix. The images also clearly show a heterogeneous distribution of W and the pore formation inside or near the W zones. Only a few (sub-)micrometer pores are visible in the Cu matrix directly.

### 4.7. X-ray Fluorescence Analysis of the Cylindric Specimens

Table 3 shows the measured weight percentages of the respective elements and indicates the fraction of the measured impurities.

No significant quantity of Si was measured in the cylindrical samples after the PBF-LB/M process. The highest concentration of W was measured for the milling process samples of 4.6 wt% and 4.5 wt%. For the co-injection samples, a W content of 3.4 wt% and 2.3 wt% was detected.

**Figure 11:**
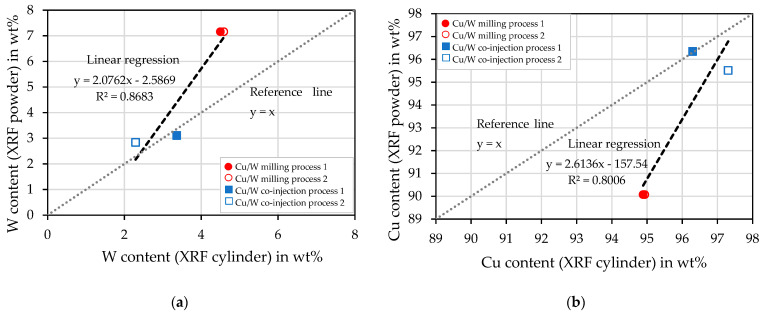
Deviations of the XRF measurements of the powder and the cylindrical sample. (**a**) W content and (**b**) Cu content.

The graphs in Figure 11a,b demonstrate the deviations between the XRF measurements of the powder and the additively manufactured sample. In addition to the linear regressions, the graphs also contain reference lines y = x to illustrate the deviations. Contrary to the expectation that Cu evaporates, reduced wt% of W are measured. The co-injection process samples show a lower deviation than the milling process samples of the measured W content.

### 4.8. Relative Density of the Cylindrical Samples

The results of the two applied methods for determining the relative density, i.e., an optical evaluation of the porosity on micro-sectioned surfaces and the density measurement according to the Archimedes principle, are listed in Table 4.

For both measurement methods, the Cu reference (raw material) sample has a relative density of 100%. Of the additively manufactured samples, the Cu/W milling process samples have the highest relative densities, followed by the Cu/W co-injection process samples. The additively manufactured Cu sample has the lowest relative density. Measured according to Archimedes, the relative density is 9.5% lower compared to the Cu/W milling process sample. The results show that a higher relative density is determined through the optical measurement.

**Figure 12:**
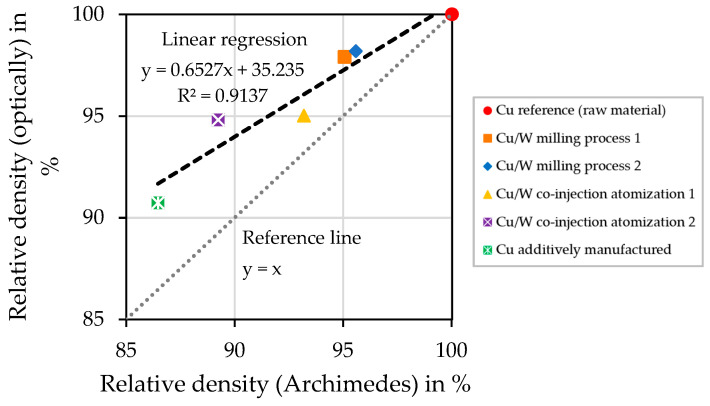
Comparison between optically and via Archimedes principle determined relative densities.

The graph in Figure 12 compares the optical and Archimedes measurement methods for determining the relative density. The deviation of the gradient illustrates the measurement discrepancy. The deviation increases with a higher porosity. To visualize the discrepancy, a reference line y = x is attached to the graph. The reasons for the deviations can be that a part of small gas porosity is not detected by the optical method. Furthermore, pores can be clogged during sample preparation due to the high ductility of Cu, which makes optical detection impossible.

**Figure 13:**
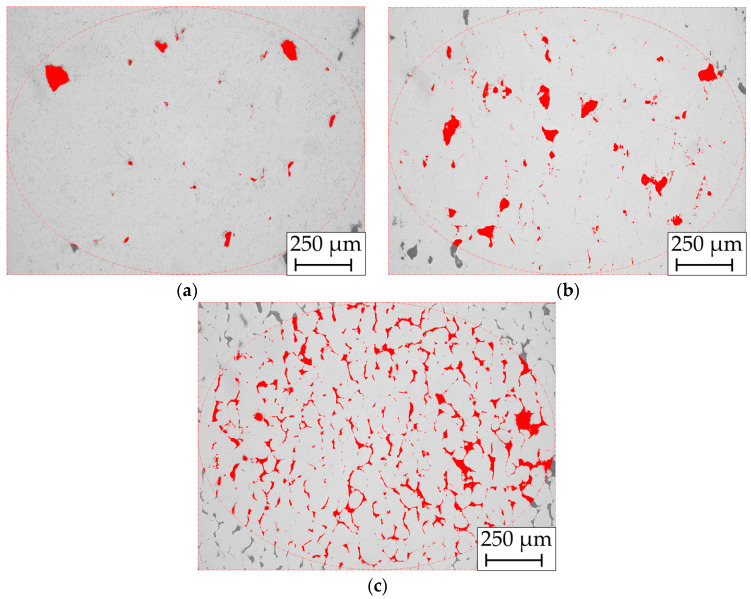
Optical porosity measurement of the cross-section of (**a**) Cu/W milling process 1, (**b**) Cu/W co-injection atomization 1, and (**c**) additively manufactured Cu.

In Figure 13, the microstructures of the optical porosity measurement are shown. Hereby, different grades of porosity Φ are depicted. Figure 13a shows the Cu/W milling process 1 sample (Φ = 1.31%).

In Figure 13b, the Cu/W co-injection atomization 1 sample (Φ = 3.52%) and (c) additively manufactured Cu (Φ = 9.31%) sample are depicted. They show irregularly shaped porosity, identified as a lack of fusion porosity. The ROI (region of interest) function is used for this, which limits an ellipse-shaped area that is used to calculate porosity. Thereby, the examined area is surrounded by a red dashed circle. The difficulty arises in selecting the contrast coverage factor to fill the entire pores without occupying any areas in the dense microstructure.

**Figure 14:**
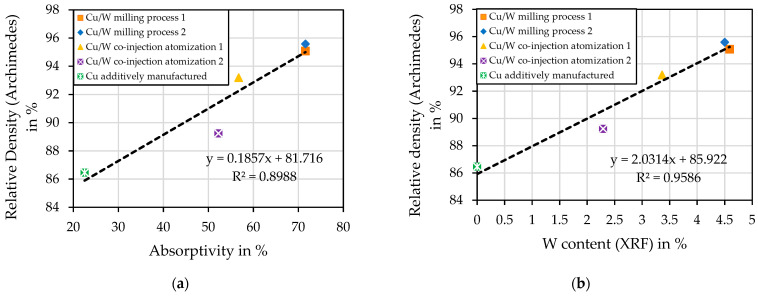
(**a**) The influence of the absorptivity of the powders and (**b**) the W content on the measured relative density of the additively manufactured test specimens.

Figure 14a shows the effect of absorptivity on the relative density of the additively manufactured test specimens. By increasing the absorptivity of the powder, more laser energy is absorbed by the W particles and is transferred by heat conduction to the Cu particles, which then melt and form the melt pool. The Cu particles themselves are also able to absorb laser energy, as the particle shape scatters the light. Figure 14b shows the influence of the W component on the relative density, which also proves the assumption that the W content enables the processability of Cu.

### 4.9. Thermal Conductivity—Laser Flash Analysis (LFA) and Thermo-Optical Measurement (TOM)

**Figure 15:**
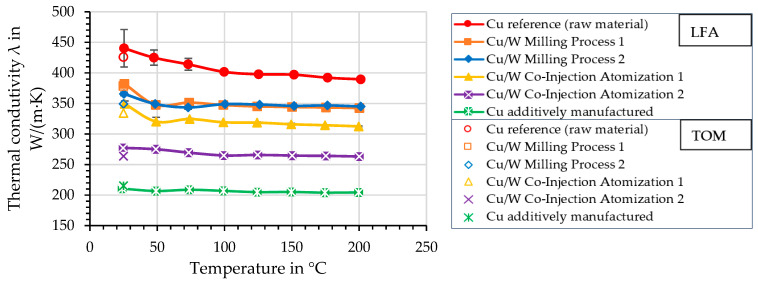
Thermal conductivity as a function of the temperature was measured by the laser flash analysis and thermo-optical measurement.

Figure 15 shows the thermal conductivity as a function of temperature. The Cu reference (raw material) sample shows the highest thermal conductivity with 440.4 W/(m·K) with a high error value of +/− 31 W/(m·K).

This is 40.4 W/(m·K) higher than the literature value of 400 W/(m·K) [28]. It should be noted that the initial temperature is 25 °C instead of 20 °C as given in the literature. In addition, there is a poor signal–noise ratio, which can lead to a high error value during measurement. With increased pulse energy, the signal is also amplified. However, the laser pulses remove the graphite layer that is necessary to absorb the laser pulses. The temperature range for the measurements is between 25 and 200 °C. The Cu/W milling process specimens show the highest thermal conductivity of the Cu/W composites with 365.8 and 382.0 W/(m·K), which is up to 86.7% of the Cu reference sample. The co-injection samples have a lower thermal conductivity of 277.3 and 349.2 W/(m·K). The additively manufactured Cu sample has a significantly lower value, with a thermal conductivity of 210.6 W/(m·K). Heat transport in a metal depends on free electrons and phonons caused by lattice vibration but is dominated by free electrons. Dissolved atoms and defects can result in electron scattering, which is related to a reduction in heat transfer [41]. Even though the samples made of powder from the co-injection atomization process contain less W than the milling process samples, they exhibit a lower thermal conductivity due to their higher porosity. Compared to a dense component, less heat flow is dissipated over a reduced cross-sectional area. In general, TOM results have a lower deviation compared with the literature values. Therefore, the results of the TOM were used for comparative observations in the following.

### 4.10. Electrical Conductivity—Eddy Current Measurement

**Figure 16:**
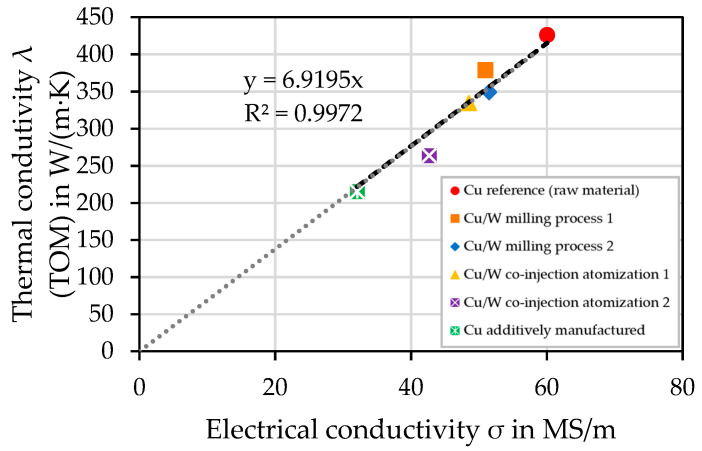
Thermal conductivity (TOM) plotted against the electrical conductivity.

Figure 16 shows the thermal conductivity as a function of the electrical conductivity determined by the eddy current measurement. The graph (dashed line) was calculated as a linear function with an intersection through the origin, depicted by the dotted line. The high correlation coefficient (R^2^ = 0.9972) implies a correlation between thermal conductivity and electrical conductivity. The Cu sample (reference) has the highest electrical conductivity of 60.0 MS/m. The literature value for Cu is 58.0 MS/m according to the International Annealed Copper Standard (IACS). Therefore, the measured value is approximately 3% higher. The Cu/W composites with the highest electrical conductivity were the milling process samples, which achieved an electrical conductivity of 50.9 MS/m and 51.5 MS/m. This is 88.8% compared to the literature value and 85.8% in comparison to the measured Cu reference sample. The co-injection samples have an electrical conductivity of 42.7 MS/m and 48.5 MS/m, which is on average 11% lower than the milled process samples. The additively manufactured Cu sample had the lowest electrical conductivity of 32.0 MS/m. In the additively manufactured Cu sample, the measured electrical conductivity value was 55.2% according to IACS.

The cross-sectional area is also crucial for electrical conductivity. This area will be reduced by pores. The same number of electrons must move through a reduced cross-sectional area. Thus, the resistance of the material is increased and the electrical conductivity is reduced. The transmission of electric current through a metal (conductor) is influenced by the same factors as the heat flow, since similarly to heat conduction, electrical conductivity is proportional to the concentration of electrons and their mobility [41].

### 4.11. Influence of Porosity on Thermal and Electrical Conductivity

**Figure 17:**
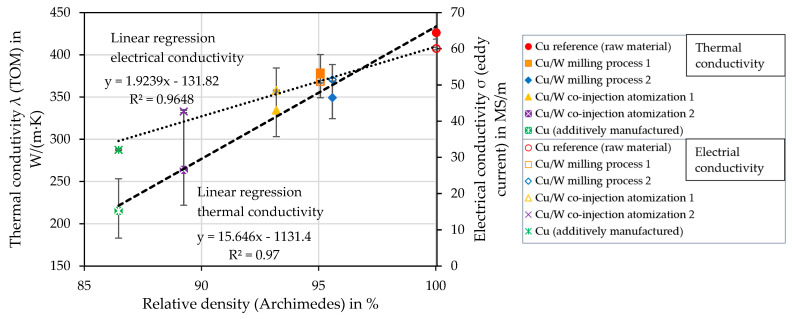
Dependence of relative density on thermal and electrical conductivity.

Figure 17 plots thermal conductivity (TOM) and electrical conductivity (eddy current measurement) as a function of relative density. In addition to the measured values, the linear regressions of the electrical conductivity (dotted line) and the thermal conductivity (dashed line) are also included. Both conductivity properties depend highly on the relative density (Archimedes) of the test specimen. With increasing relative density, higher thermal conductivities and electrical conductivities are measured.

Pores and W zones reduce the Cu cross-sectional areas and thus reduce the mobility of electrons and the transfer of phonons. Due to the small W content and its insolubility, it has a low effect on conductivity properties. Furthermore, a heterogeneous distribution of W could lead to barrier layers, which could influence the conductivity properties [42]. However, the W content is not high enough for this either. In contrast, the absorptivity of the W particles is essential for the processability of the material and thus reduces the lack of fusion porosity. The present results indicate that porosity is the primary factor affecting the conductivity properties.

### 4.12. Maxwell Model of Thermal Conductivity of MMCs

In the study of Koltsova et al. [43], the Maxwell equation is used to calculate the effective thermal conductivity of an MMC. Kiradjiev et al. [44] apply the Maxwell model to calculate the thermal conductivity of a porous material. In the present study, the Maxwell equation is used to predict the thermal conductivity of a porous MMC.

(3) $$\frac{k_{eff}}{k_{m}}=1+\frac{3\varphi }{\left(\frac{k_{1}+{2k}_{m}}{k_{1}-k_{m}}\right)-\varphi }$$

k_eff_ = effective thermal conductivity of the composite material.

k_m_ = thermal conductivity of the matrix.

k_1_ = thermal conductivity of the filler particles or pores.

*φ* = volume fraction of the filler particles or pores.

The Maxwell model assumes a continuous matrix surrounding the spherical filler particles. Furthermore, no interaction between the filler particles is considered. In addition, the model is valid for <25% volume fraction of the filler particles [45].

**Figure 18:**
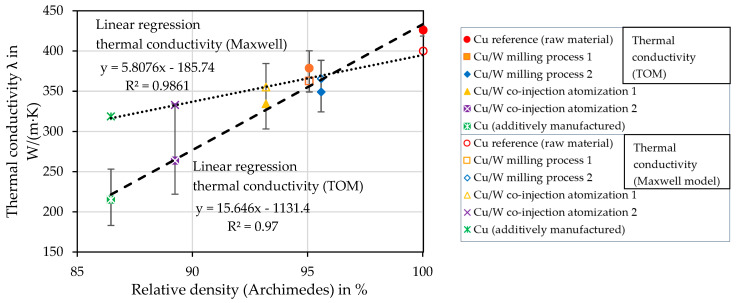
Comparison of the thermal conductivities (TOM) and the theoretical calculation (Maxwell equation) as a function of the relative density (Archimedes principle).

The thermal conductivity of the dense Cu/W composite is calculated as follows. The thermal conductivity for the matrix (Cu) is k_m_ = 400 W/m∙K [28] and for the filler material (W) k_1_ = 175 W/m∙K [29]. The respective W volume fraction is used for the volume fraction of the filler material *φ*. For calculating the thermal conductivity of the porous Cu/W composites, the calculated k_eff_ for the dense Cu/W composites is used for k_m_. For k_1_, it is assumed in simplified form that no heat flow occurs via the pores. Therefore, k_1_= 0. The porosity determined according to Archimedes is used for the volume fraction *φ* of the voids. In Figure 18, the measured and theoretically calculated thermal conductivities are shown in the graph as a function of the relative density. The linear regressions of the thermal conductivity calculated by Maxwell’s model (dotted line) and the thermal conductivity measured using TOM (dashed line) are plotted in the graph.

The measured specimens Cu reference and Cu/W milling process 1 have a higher thermal conductivity than the theoretical calculation because of the measurement failure, as mentioned in Section 4.9. Another influence could be the presence of alloying elements, which reduce the conductivity properties. The main reason for the discrepancies in the measurement results compared to the theoretical calculation is defects, i.e., gaps and cracks, which reduce the thermal conductivity but are not included in the calculation. This is especially shown for the samples with increased porosity. In addition, the sphericity and homogeneous distribution of the voids and filler particles are not given.

### 4.13. Hardness—Vickers Measurement

**Figure 19:**
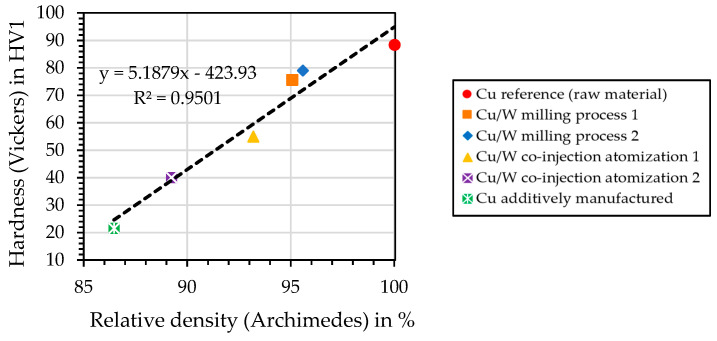
Influence of relative density on the hardness.

The influence of relative density on hardness is shown in Figure 19. The highest hardness was measured for the Cu reference material. The Cu/W milling process specimens have the highest hardness of the additively manufactured samples. The Cu/W co-injection samples have a reduced hardness. The additively manufactured Cu sample shows the lowest hardness. At low W content, the hardness correlates with the relative density. The plastic ductile matrix of Cu can be pressed into the pores by the acting force. As a result, the material to be tested deforms more with higher porosity, and a lower hardness is achieved. The Cu/W composites do not contain enough W to strengthen the material through the W zones.

### 4.14. Demonstrator

**Figure 20:**
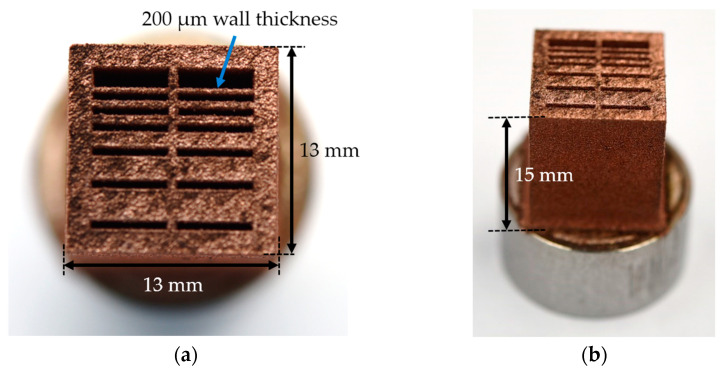
(**a**,**b**) Cu/W composite component with varying wall thickness.

To prove the qualification of the powder for the PBF-LB/M process, a component with different wall thicknesses was built. The component with a geometry of 13 × 13 × 15 mm is illustrated in Figure 20a,b.

With the Cu/W composite powder and optimized process parameters, wall thicknesses down to 200 µm can be produced. This also demonstrates the respective applications of the additively manufactured material, for example, as a heat exchanger, heat sink, or coil.

## 5. Conclusions

To summarize, co-injection atomization offers a cost-effective alternative to the additional milling process. There are also other advantages, such as the prevention of contamination and oxidation. Furthermore, the Cu particles can be coated without being deformed, which positively affects their flowability. However, there are disadvantages, such as limited W fractions. Moreover, achieving the complete surface coverage of the Cu particles with the fine W particles is difficult without forming an increased number of W agglomerates in the Cu/W composite powder. Finally, controlling the exact composition of Cu and W poses a challenge.

In comparison to the Cu reference (raw material), the Cu/W composite from the milling process has a thermal conductivity of 88.9% and an electrical conductivity of 85.8%. If the Cu/W composite is compared to the additively manufactured Cu sample, the relative density increases by 10.5% (Archimedes). Due to this decrease in porosity, identified as the governing factor for impeding thermal and electrical conduction, an increase in the thermal conductivity of 81.4% and in the electrical conductivity of 60.9% is achieved. The W zones represent only 3 vol% of the composite in the milling process samples. Consequently, the negative effect of W zones on the conductivity properties is limited. However, the W particles absorb the electromagnetic radiation from the laser and transfer the resulting thermal energy to the Cu particles through heat conduction. This enables a reduction in pores in the manufactured Cu/W composite and thus directly improves the processability of the composite powders. In addition, a demonstrator component of the Cu/W composite of different wall thicknesses shows the potential for thermal and electrical applications.

Current research activities are concerned with optimizing the W content to optimize material properties. Further investigations into the long-term stability of Cu/W components to improve corrosion resistance and thermomechanical stability are also planned.

## Figures and Tables

**Table 1:** Apparent and tap density of the powders.

Powder Sample	Apparent Density in g/cm^3^	Tap Density in g/cm^3^
Cu	4.32	4.73
W	2.38	4.09
Cu/W milling process	4.62	5.45
Cu/W co-injection atomization 1	4.54	5.19
Cu/W co-injection atomization 2	4.41	4.90

**Table 2:** Elemental analysis with the XRF of the powders.

Powder Sample	Cu in wt%	σ +/−	W in wt%	σ +/−	Impurities in wt%
Cu	99.84	0.29	-	-	0.16
W	-	-	99.66	0.54	0.34
Cu/W milling process	90.07	0.28	7.16	0.12	2.78
Cu/W co-injection atomization 1	96.35	0.26	3.11	0.08	0.55
Cu/W co-injection atomization 2	95.51	0.26	2.84	0.07	1.65

**Table 3:** Elemental analysis with the XRF of the cylindrical specimens.

Cylindrical Sample	Cu in wt%	σ +/−	W in wt%	σ +/−	Impurities in wt%
Cu reference (raw material)	99.69	0.29	-	-	0.31
Cu/W milling process 1	94.89	0.26	4.59	0.09	0.52
Cu/W milling process 2	94.94	0.25	4.50	0.09	0.56
Cu/W co-injection atomization 1	96.30	0.26	3.36	0.08	0.34
Cu/W co-injection atomization 2	97.31	0.26	2.30	0.07	0.40
Cu additively manufactured	99.60	0.29	-	-	0.41

**Table 4:** Relative densities measured optically and according to Archimedes principle.

Sample	Relative Density (Optically) in %	Relative Density (Archimedes) in %
Cu reference (raw material)	100.0	100.0
Cu/W milling process 1	97.9	95.1
Cu/W milling process 2	98.2	95.6
Cu/W co-injection atomization 1	95.0	93.2
Cu/W co-injection atomization 2	94.8	89.2
Cu additively manufactured	90.7	86.5

## Data Availability

The original contributions presented in the study are included in the article, further inquiries can be directed to the corresponding author.
